# A perspective on the role of language about pain

**DOI:** 10.3389/fpain.2023.1251676

**Published:** 2023-08-02

**Authors:** Simon van Rysewyk

**Affiliations:** Department of Philosophy and Gender Studies, School of Humanities, University of Tasmania, Hobart, TAS, Australia

**Keywords:** pain, Wittgenstein, language, rule, rural

## Abstract

This article contributes a perspective on pain motivated by the philosopher Ludwig Wittgenstein. According to Wittgenstein, the child learns from others that the occasions on which it manifests certain reactions—the reactions that human beings manifest when injured—make it appropriate to self-ascribe “pain”. When the child can signal correctly that she is in the requisite bodily state, then she has a conception of pain. Using the concept *pain* to symbolise an experience also makes it possible to tell other people what is going on and to solicit their help in managing the pain. In pain discourse, we can say “Sam can tell that Jason is with pain”, or “She could tell you that Jason is with pain if she wanted to”. These uses are linked to social milieu where rules are learnt for the application of concepts, such as the concept *stoicism*. In many rural communities, adults tell other adults about pain when it interrupts work or social activities. Otherwise, it is normative to “carry on”. The rural stoic who tells another about pain only if he wants to can complicate clinical pain management, which can undermine the patient's special authority. In contrast, convergence in pain definitions and judgements between the patient and health professional can protect the authority of the patient and improve the clinical interaction. Pain is not simply a quale that is privately perceived; it must be capable of being expressed. Thus, pain has a social role, which is learnt. The study of linguistic rules in pain discourse could help explain the learning and application of the concept *pain*.

## Introduction

A significant body of knowledge exists on the pathologies, injuries, and diseases, as sources of pain, and their impact on the host, as related to neurobiology and personal experience. The contribution of the social environment in which pain is experienced has received less study perhaps because we think of the personal experience as given, and the behavioural manifestation of the experience as secondary, as symptoms through which we come to know the mind of another human being. Pain is undoubtedly a private experience but ascribing the word “pain” to other human beings presupposes the possibility of behavioural manifestations (1–3). Behavioural manifestations of pain include facial expressions and bodily gestures, what people say and do, and the occasions for the use of “pain” (4). If we encountered a society of people who used a word that lacked any connection with pain-related behaviour, and the complex situations in which we show pain, we would not translate it as “pain”. Pain is neither totally reducible to, nor totally separable from, its associated behaviours. Pain must be capable of being expressed (3).

Understanding pain means recognising that it is an event that occurs in the weave of social life, with the person's pain manifestly obvious, usually based on the social context and reactions of others. In a community of people who manifest pain behaviour as we do, but do not react to it with sympathy, “pain” would not have the same meaning. The two communities could be more in harmony if the social context is used to make sense of some phenomena connected to pain, such as “disinterest in other people with pain”, although this couldn't hold for some pain-behaviours, notably involuntary manifestations of pain like facial expression or paralinguistic speech, as phenomena like apathy, hope, pretence, or expectation are not assigned to others using straightforward behavioural criteria but require an elaborate social context (3).

Linguistic skills are learnt in specific familial and ethnic contexts and provide the child a symbolic mode of reacting to “what is going on”. The child learns from others that the occasions on which it manifests certain reactions make it appropriate to self-ascribe “pain”. This step instructs the child what “pain” refers to. This skill develops into more sophisticated linguistic abilities, which include using more complex statements about pain (“Her pain is getting worse”, “The dog is howling with pain”, “He is not in pain now”), and relating pain to other experiences (“I am not in pain; I’m just sad”) and to reason-governed action (“Going to the dentist will help my toothache”) (3, 4). When the child can tell that it is with pain, it not only can register a private experience, but it can also give the *concept pain* a role in social activity and in the organisation of social reactions. According to Wittgenstein, it is a mistake to think that we can meaningfully speak about things that are experienced *only* in the private mind (3). Consequently, if mental contents are not only meaningfully private, then there is an essential link between personal experiences like pain and the social world. In the next sections, I briefly survey the elements of Wittgenstein's argument, focusing on linguistic meaning, rules of language, and agreement in judgements. Following this review, I apply these elements to the rule-governed use of concepts such as *stoicism* in rural pain discourse.

### A brief survey of Wittgenstein's “private language argument”

#### Linguistic meaning is use

Wittgenstein claims that understanding what a word means involves correct (normative) use: “For a large class of cases—though not for all—in which we employ the word ‘meaning’ it can be defined thus: the meaning of a word is its use in the language” (3, §43). Thus, in the following clinical anecdote, it is unclear if the patient understands the word “pain”: “After the operation, the patient no longer complained spontaneously of pain and no longer appeared to be in distress, though when asked, he acknowledged that pain was still present” (5, p. 588). If the ward nurse checks the patient's understanding, she could ask, “Does your pain bother you?” If the patient does not demonstrate real-life familiarity with the use of “pain” or does not do anything that normally is inseparable from feeling pain, the nurse could infer that he does not understand it (or the situation is too ambiguous to resolve). For Wittgenstein, understanding is correct use, which is a social fact.

#### Rules of language

The person who can self-ascribe “pain” can correctly discriminate reactions that make it appropriate to say, “I feel pain”, from reactions that do not. Thus, the person who understands the concept *pain* can understand and communicate pain, and only pain, by use of the concept. For example, the person who understands the concept pain:

• Does not self-ascribe “pain” to sensations of hunger or thirst.
• Does not self-ascribe “pain” based on inference; for example, using introspection, categorisation, behavioural observation, or verifying the cause of the pain.
• Does not misidentify who is with pain; for example, she does not claim “I believe my pain was Eli's, not mine, although I can’t be sure”.

Correct use of “pain” means following rules that link pain and the word “pain”. In practice, linguistic mastery of “pain” is achieved by using the word such that the person's successes and errors are in principle manifest and can be corrected by others (3, §202). The successful learner shows enough correct application to count as having understood the concept *pain*. Wittgenstein concludes, “Hence it is not possible to obey a rule ‘privately’: otherwise thinking one was obeying a rule would be the same thing as obeying it’’ (3, §202). The truth of privacy in relation to pain is not introspective knowledge, but that what I say or utter about my pain is a spontaneous and authentic manifestation of “what is going on within me”. The special role granted the person with pain is not a special knower, but a special actor (6).

#### Agreement in linguistic judgements

Learning the concept *pain* is triangulated with interpersonal interactions and behaviour in which personal judgements about pain in oneself and others are manifest to others and corrected by them. The person who achieves a sufficient level of agreement in judgements with others is counted as having mastered the rule-governed ability use of “pain”. The result of this educational training is that judgements of the learner gradually converge with those of others who already understand the concept *pain*. In the clinical situation quoted above in which self-ascribed pain does not lead to agreement in judgements, the patient's care team must determine whether the patient understands what pain is. The conclusion the team arrives at would rely on the patient's use of “pain” in different situations, and the role he or she gives the concept in a range of thoughts.

The possibility of disagreement in judgements about the pain of others reflects an indeterminacy, which is constitutive of our concept *pain* (3). That indeterminacy in turn is due to social patterns of behaviour: our concept *pain* must be flexible because pain behaviour, and our complex reactions to it, is diverse and unpredictable (3, 4). Caregiving in pain settings can involve a threat of pain to the carer and is conditional on the authenticity of manifest pain behaviour. As observers of pain in other people, we are sensitive to signs of exaggeration, suppression, or malingering, in behavioural displays of pain (4). Accordingly, our concept *pain* does not always rigidly connect behaviour, situation, and personal experience (3). Given the same evidence, one health professional can be convinced of a diagnosis, another is not (7). However, we do not on account of this disagreement exclude either from the medical profession, as being unaccountable or incapable of judgement. This reflects not professional incompetence, but the indefiniteness of pain.

## Discussion

### Telling others that I am with pain

Using the concept *pain* to symbolise an experience makes it possible to tell other things about it. For example, “I feel better now”, “It burns”, “The pain is spreading”, “I need a break”. It also allows a person to tell other people what is happening and to solicit their help in delivering analgesia and care. However, in the swing and play of life, people tend to be careful about who and when they communicate pain (4). The unpredictability of social reactions to pain in others reveals indeterminacy in our concept *pain*, which is reflected in different uses of “tell”. In pain discourse, “telling” can be used in at least two ways. We can say “Eli can tell that Susan is with pain”, or “He could tell you that Susan is with pain if he wanted to”. Again, these uses are linked to participation in a social milieu where specific rules are learnt for the regulation of concepts. For example, people growing up and living in rural communities in Australia and New Zealand conventionally learn social rules for the use of certain concepts, commonly self-reliance, stoicism, or fatalism (8–11), which make them more accepting of chronic pain, disease, or illness than people in urban environments. These concepts are paired with rules that instruct when it is appropriate to tell others about being with pain. A common rule in rural communities is for adults to tell other adults about personal pain when it interferes with work or important social activities (12). Otherwise, it is appropriate to “carry on” (8). One farmer in New Zealand observed (8, p. 403):

“Our home is where our farm is, where it's all encapsulated together. So, our whole family, the whole structure is—maybe not for all farmers, I don’t think that's true, but for many of us. So, we don’t just look for what's gratification for ourselves, we’re looking through for the next generation, wanting to provide. So that's why we struggle with removing ourselves from the workplace”.

Who in particular is told about pain in rural areas is also guided by rules. In rural and remote Australia, the GP in some communities is from a different culture and speaks English as a second language (13). Rural people tend to distrust “outsiders”, including medical professionals, consulting instead with their own community networks for assistance or advice about pain or illness (14, 15). This practice could mean that rural people prefer concepts to function against a more stable than changing background; therefore, concepts must be more determinate than indeterminate. Such speculation highlights the importance of tight kinship in rural community networks, for correct use of the concept *pain* involves convergence in judgements about what is significantly the *same;* thus, it involves understanding the consequences of pain in the rural milieu. Telling another about being with pain involves having some idea what to expect from the other and being able to relate to and understand this person. Thus, together with applying certain rules, “telling” in this setting also involves a sensitivity, or “feel” for human behaviour. This could explain why rural people prefer not to tell “outsiders” about personal pain.

“Well, the one before Dr P. didn’t understand, just didn’t know anything about this and wasn’t interested. He just said straight out that wasn’t his line—he wasn’t going that way. And he couldn’t understand, I suppose, the amount of pain. He was forever telling me that ‘don’t do this, don’t do that’. In his opinion I should have been just be sitting in my chair you know, knitting the rest of my life away” (16, p. 481).

Telling another about being with pain *if one wants to* can create divergence between patient verbal self-report and non-verbal pain behaviour, which can limit or obstruct the efforts of health professionals to intervene on the patient's behalf, resulting in inadequately managed pain (17). In comparing rural and urban nursing homes, rural nursing home staff, “more so than their urban counterparts, emphasized stoicism as an attitudinal barrier on the part of residents that interfered with pain assessment” (18, p. 745). A foundation of pain discourse is that the person's sincere utterances about his or her own pain are treated as correct. To introduce doubt here (e.g., “stoicism as an attitudinal barrier”) could alter normal discourse; specifically, it could undermine the authority of the subject. In contrast, convergence in pain definitions and judgements can preserve the special role of the person with pain and improve the clinical interaction. In a qualitative ethology study (17), Spiers describes a rule co-created between patients and nurses in urban home-based care, for which “stoicism did not imply enduring excessive pain but…the ability to know where one's pain boundaries lay and to take appropriate measures to keep pain within those boundaries” (17, p. 296). The nurses effectively implemented this shared rule using different communication strategies (17).

From clinical cases like these, Beeckman et al. (18) speculates that rules in the pain setting are a “double-edged sword” as rigidly following pain-related rules despite costs could be a risk factor for worse outcomes, whereas flexibly switching between pain-related rules—e.g., exchanging a rule stipulating pain-related avoidance for one stipulating acceptance of pain—depending on the situation and benefits could help explain “resilient functioning” with chronic pain. According to rural nurses, generational and geographical factors explain stoicism in rural people with chronic pain (19). Residents in rural nursing homes expect to be with pain; according to nurses, “It's how they age here” (19, p. 745). For rural residents with pain, the switch to following a new pain rule would need to contribute to desired consequences (e.g., minimal interruption to work) but perhaps more importantly, the new rule would need to integrate into the fabric of rural life: “…we don't just look for what's gratification for ourselves, we’re looking through for the next generation, wanting to provide” (8, p. 403). As our needs or interests change, or simply as part of life, our concepts can evolve over time. An evolved concept could supply us a new rule guiding the way we behave, which could correspond more or less with existing rules (3). Although our concept *pain* is flexible on the rules we use, the rules *we* adopt must be usable.

In experimental studies, rural groups tend to report higher rates and more intense chronic pain than people from urban groups (e.g. 20),. Poorer access to pain treatments for rural compared to urban residents could partly explain differences in pain outcomes (e.g. 21–23),. Another possibility is that urban residents are less attentive to their pain due to the multiple interruptions that can compete for their attention in the city environment (20). By contrast, rural living is described as “peaceful, tranquil, spacious, friendly and caring” (24, p. 211). With fewer external interruptions competing for personal attention in the rural setting, rural residents could have learnt, on average, to be more alert to pain and its qualities than urban residents.

In this article, I have argued for the perspective that pain is not merely a “raw feel” that is privately and unproblematically perceived by human beings; it also has a social role, which is learnt. When a person can correctly signal that she is in the requisite bodily state, then she has a conception of pain. The risk of disagreement in our judgements about others with pain, which rests on our diverse and unpredictable reactions to pain behaviour, should motivate trust-building and shared decision-making in the clinic. I have lent support to these claims in the article through linking the ambiguity of “tell” to rules for the use of concepts, such as *stoicism*, as applied in rural settings.

## Data Availability

The original contributions presented in the study are included in the article/Supplementary Material. Further inquiries can be directed to the corresponding author.
